# A Safety Reinforced Cooperative Adaptive Cruise Control Strategy Accounting for Dynamic Vehicle-to-Vehicle Communication Failure

**DOI:** 10.3390/s21186158

**Published:** 2021-09-14

**Authors:** Yi Liu, Wei Wang

**Affiliations:** 1 Jiangsu Key Laboratory of Urban ITS, Southeast University, Si Pai Lou #2, Nanjing 210096, China; dlmu_liuyi@163.com; 2 Jiangsu Province Collaborative Innovation Center of Modern Urban Traffic Technologies, Southeast University, Si Pai Lou #2, Nanjing 210096, China; 3 School of Transportation, Southeast University, Si Pai Lou #2, Nanjing 210096, China

**Keywords:** connected vehicles, vehicle platoon, CACC, communication failure

## Abstract

Cooperative Adaptive Cruise Control (CACC) is an advanced technique for organizing and managing a vehicle platoon, which employs the Vehicle-to-Vehicle/Vehicle-to-Infrastructure (V2V/V2I, or V2X) wireless communication to minimize the inter-vehicle distance while guaranteeing string-stability. Consequently, the conventional CACC system relies heavily on the quality of communications, which means that the regular CACC platoon is sensitive to the communication failure. Therefore, in this paper, a Safety Reinforced Cooperative Adaptive Cruise Control (SR-CACC) strategy is proposed to resist unexpected communication failure. Different from the regular CACC system, the safety enhanced platoon control system is embedded with a dual-branch control strategy. When a fatal wireless communication failure is detected and confirmed, the SR-CACC system will automatically activate the alternative sensor-based adaptive cruise control strategy. Moreover, to make the transforming process smooth, a linear smooth transition algorithm is added to the SR-CACC system. Then, to verify the performance of the proposed SR-CACC system, we conducted a simulation experiment with a heterogonous platoon constructed with eight vehicles. The experiments results reveal that, under the extremely poor communication environment, the proposed SR-CACC strategy can significantly improve the safety performance of the organized vehicle platoon.

## 1. Introduction

As the World Health Organization (WHO) revealed in the 2018 global road traffic accident report [1], road traffic crashes result in 1.35 million annual fatalities and leave 20 and 50 million people suffering from non-fatal injuries around the world. The past two decades have seen an increase in the number of traffic jams and accidents. Survey and statistical analysis have revealed that, of all the traffic accidents that have ever taken place, about 60% to 70% were caused by vehicular collisions, especially rear-end collisions [2]. In addition to inflicting pain and suffering, traffic accidents also impose a heavy economic burden on victims and their families, both through treatment costs for the injured and through the loss of productivity of those killed or disabled [3]. The direct economic costs of global road crashes have been estimated at US$ 518 billion, which is about 1.5% and 2% of the annual Gross National Product (GNP) of middle-income countries and high-income countries, respectively [4]. Road traffic injuries constitute a major public health and development crisis and are predicted to increase if road safety is not addressed adequately by administrations. Nevertheless, road safety is an issue that does not receive the attention it deserves—and it is one of our great opportunities to save lives around the world [5].

Though the causes of traffic accidents are complex, most road traffic injures are caused by rear-end collisions. From previous research, it was discovered that driver operation errors are one of the major causes of road collision accidents [6,7]. Approximately 94% of traffic accidents and fatalities are mainly caused by human factors, such as drowsiness, distractions, or improper decisions [8]. Furthermore, in traffic accidents caused by inattentiveness, almost 50% of them are directly triggered by drivers’ distractions. In order to reduce drivers’ physical and mental burden, both academia and industry have shown great interest in seeking robust autonomous vehicles to substitute the error-prone human driving [9,10]. Therefore, developing a reliable autonomous vehicle control system that replicates the desired driving behaviors to avoid traffic collisions is still an ongoing research challenge [11]. 

In recent years, with the development of advanced sensors, such as optical sensors (camera), LiDAR (Light Detection and Ranging), ultrasonic Radar (Radio Detection and Ranging), and millimeter-wave Radar, a new autonomous driving era has been inaugurated. However, due to the intrinsic limitations of these vehicle-mounted sensors, autonomous vehicles are prone to making erroneous decisions, eventually leading to serious accidents [11]. For example, on 1 March 2018, a Tesla Model 3 crashed into the side of a truck on a Florida highway, killing the driver on the spot, which was at least the third fatal U.S. crash accident involving the driver-assistance system, as the National Transportation Safety Board reported [12]. In addition, another incident occurred on 19 March 2018, although the sensors equipped on Uber’s autonomous car detected the pedestrian, the vehicle executed the wrong decision, leading to the tragedy of a 49-year-old woman’s death [13]. Therefore, for enhancing the safety level of road transportation, it is not adequate to simply improve the performance of individual vehicles.

**Figure 1:**
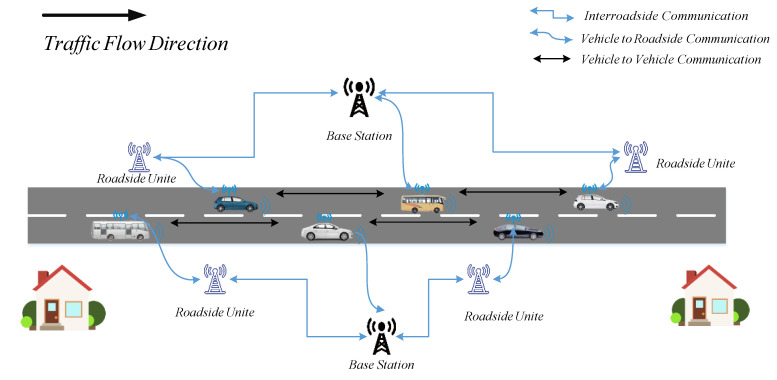
Illustration of Vehicular Ad hoc NETwork (VANET) for autonomous vehicles.

At this point, V2V and V2I wireless technologies facilitate the application and development of a cooperative autonomous vehicle platoon, which can somewhat make up for the deficiency of the individual self-driving system. As shown in Figure 1, the Vehicular Ad hoc NETwork (VANET) systems expand vehicles’ capabilities through the integration of computation, communication, and automatic control [14]. Based on wireless communication, VANET, and physical processes, a vehicle platoon is an important vehicular cyber-physical (CPS) application. Furthermore, Cooperative Adaptive Cruise Control (CACC) is one of the most effective ways to organize vehicle platoons, and seems to be more reliable, feasible, and efficient to promote inter-vehicle information exchange [11]. 

In recent years, cooperative adaptive cruise control has been a hot topic in intelligent transportation research. In previous research, cooperative adaptive cruise driving has been widely studied, in terms of different aspects, such as fuel consumption [6], homogeneity [7] or heterogeneity [3,8] of teams, and vehicle spacing policies over time [9] or space [4], among others. Some research focused on the environmental benefits of autonomous vehicles platoons, the work in [15] reveals that a CAV platoon can greatly reduce vehicle fuel consumption and emissions. Moreover, various information flow topologies (IFTs) have been proposed in previous works in the literature, e.g., one-look-ahead, two predecessors following, leader-following, leader-predecessor following, multiple-look-ahead, and multiple predecessors and followers [14]. With perfect communication networks, the intervehicle distance of the CACC platoons can be minimized. As a result, traffic throughput is increased while maintaining a sufficient level of safety [16].

Consequently, the CACC system relies on the quality of communications more heavily, which means that a regular CACC platoon may be sensitive to communication failures, such as communication time delays and dropouts. To date, most of the previous research investigated CACC platoons that were mainly based on the assumption that communication networks are perfect. However, it is difficult to maintain perfect quality and excellent reliability of wireless communications, which may be affected by many unexpected impairments, such as channel fading, shadowing, and interference [17]. Therefore, the possibility of communication failure is inevitable in real vehicular communication networks, which stimulates further research into the development of safety-enhanced CACC systems to resist communication failures. To the best of our knowledge, existing research on the compensation strategy of communication failures is still scarce [18]. Motivated by these gaps, this paper mainly focuses on the problem of the cooperative vehicle platoon under imperfect communication environments, especially line formation and the maintenance of heterogenous CACC vehicle teams. More specifically, the main contributions of this paper can be concluded as:

(i) A novel dual-branch safety-reinforced cooperative adaptive cruise control strategy that is proposed to guarantee the safety of a heterogenous platoon under extremely poor communication environments.

(ii) A linear smooth transition algorithm is imposed in the proposed SR-CACC system to make the transforming process between the two controlling branches smoother. 

(iii) We designed simulation experiments to analyze the performance of the proposed SR-CACC system, where the vehicle platoon suffering from vital communication failure is considered.

The remainder of this paper is organized as follows: the following section provides the preliminaries. Then, we give the system design in Section 3, where the SR-CACC system is introduced comprehensively. Thereafter, in Section 4, the simulation experiments are designed and conducted to verify the effectiveness of the proposed strategy. Conclusions and discussions are presented in the last section of this paper.

## 2. Preliminaries

In this section, we introduce the dynamic model of heterogenous platoons and the fundamental components of the cooperative vehicle string in our study.

### 2.1. Vehicle Dynamics Model

**Figure 2:**
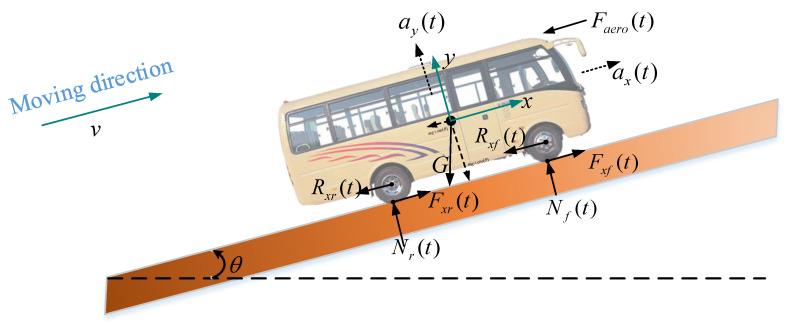
Forces analysis of a vehicle moving on an inclined road.

The cooperative motion of a platoon can be simplified as a collection of vehicles. When considering a vehicle moving on an inclined road, as shown in Figure 2, the vehicle’s longitudinal dynamics should include the engine, driveline, brake system, aerodynamics drag, tire friction, rolling resistance, gravitational force, etc. [18,19,20,21,22,23]. These external longitudinal forces acting on a vehicle can be described as follow:

From the force analysis diagram, we can establish the dynamic equations of the vehicle from the vertical (*y*-axis) and longitudinal (*x*-axis) directions, respectively. Let $x_{i}$ and $y_{i}$ denote the longitudinal and vertical position of vehicle-*i*-th on an inclined road, and $x_{i}$ increases in the forward driving direction. The vehicle dynamics equations derived from Newton’s second law can be expressed as follow [24]:

(1) $$m{\ddot{y}}_{i}=ma_{y}(t)=N_{r}(t)+N_{f}(t)-mg\cos(\theta )$$

(2) $$m{\ddot{x}}_{i}=ma_{x}(t)=F_{xr}(t)+F_{xf}(t)-F_{aero}(t)-R_{xr}(t)-R_{xf}(t)-mg\sin(\theta )$$

Equations (1) and (2) are the vehicle dynamics formulas along the vertical and longitudinal axis, respectively. Where $a_{y}(t)$ denotes the acceleration of the vehicle in the *y*-axis direction, usually its value is constant, 0; $a_{x}(t)$ is the acceleration of the vehicle in the *x*-axis direction; $N_{f}(t)$ is the vertical force at the front tires; $N_{r}(t)$ is the vertical force at the rear tires; $F_{xr}(t)$ is the longitudinal tire force at the rear tires; $F_{xf}(t)$ is the longitudinal tire force at the front tires; $F_{aero}(t)$ is the equivalent longitudinal aerodynamic drag force; $R_{xr}(t)$ is the force due to rolling resistance at the rear tires; $R_{xf}(t)$ is the force due to rolling resistance at the front tires; $N_{f}(t)$ is the vertical force at the front tires; $N_{r}(t)$ is the vertical force at the rear tires; θ is the angle of inclination of the road on which the vehicle is traveling, as shown in Figure 2, which is defined to be positive counter-clockwise when the vehicle is moving toward the right. When a vehicle is moving toward the left, the angle θ will be defined as positive clockwise.

In Equation (2), the equivalent aerodynamic drag force $F_{aero}(t)$ is directly related to the vehicle’s frontal area and real-time traveling velocity, which can be expressed as [24]:

(3) $$F_{aero}(t)=\frac{1}{2}\rho C_{d}A_{F}{\left(v_{i}+v_{wind}\right)}^{2}$$

where $v_{wind}$ represents the wind velocity, which is the projected length of the velocity of wind on the *x*-axis; $v_{i}$ is traveling velocity of the subject vehicle along the longitudinal direction; $A_{F}$ represents the frontal area of the vehicle, which is the projected area of the vehicle in the direction of travel. Table 1 summarizes the notations used in our equations for easy reference.

From Equations (2) and (3), it is evident that the vehicle dynamic model is nonlinear due to the aerodynamic drag, $F_{aero}(t)$. Fortunately, the longitudinal vehicle dynamics can be linearized using the linearization technique [25], which is employed to simplify the longitudinal vehicle dynamics in a linear form. The state of the *i*-th-vehicle can be expressed as:

(4) $$\left(\begin{array}{c} {\dot{x}}_{i}(t)\\ {\dot{v}}_{i}(t)\\ {\dot{a}}_{i}(t) \end{array}\right)=\left(\begin{array}{c} v_{i}(t)\\ a_{i}(t)\\ \frac{u_{i}\left(t\right)-a_{i}(t)}{\tau_{i}} \end{array}\right)$$

In the above linearization of the longitudinal vehicle dynamics model, $u_{i}\left(t\right)$ is the control input of the subject vehicle, which denotes the desired acceleration of the i-th vehicle at time *t*, and $\tau_{i}$ is a time constant caused by the mechanical or actuator lag, which can be different for each vehicle (*i*) determined by the performance of each vehicle’s powertrain or brake actuator. This linearized longitudinal vehicle dynamics model simplifies the complexity of the lower-level control, which enables us to focus on the upper-level cooperative platoon controller design without considering the complex underlying implementation mechanism.

### 2.2. Cooperative Adaptive Cruise Platoon Control

**Figure 3:**
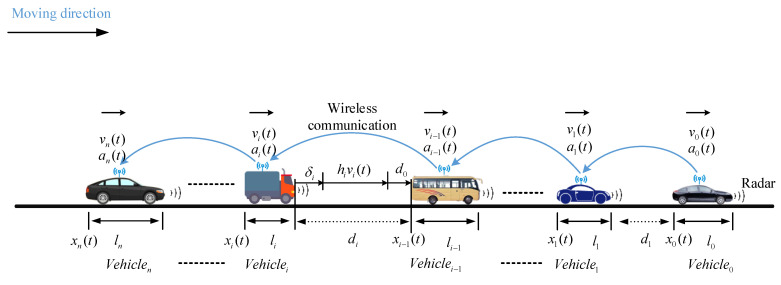
Schematic of a heterogeneous CACC vehicle platoon.

**Figure 4:**
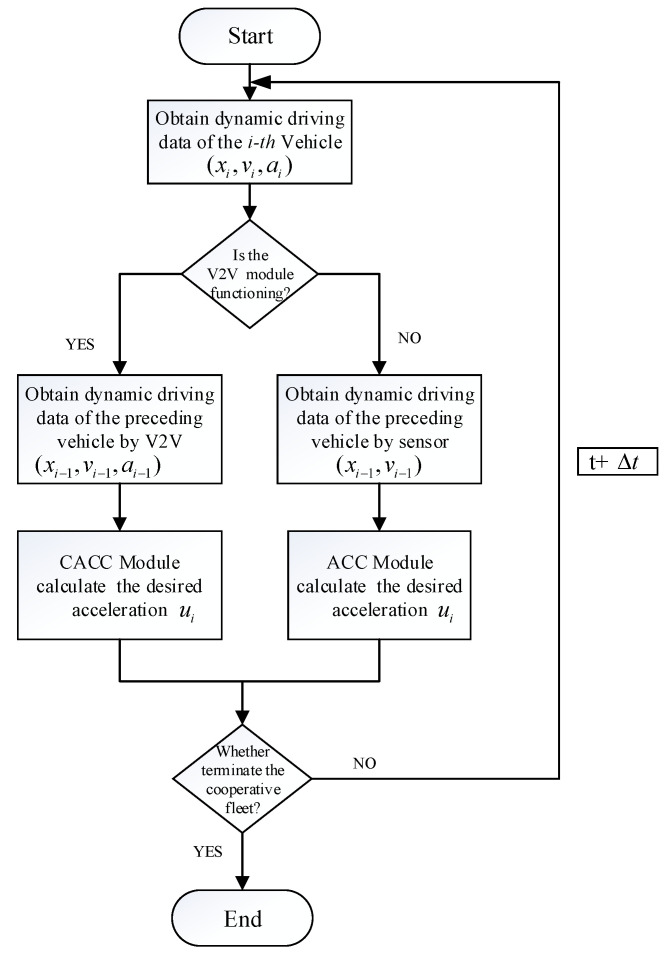
Flowchart of the safety-reinforced platoon control strategy.

Based on the introduced vehicle dynamics model, a CACC platoon with *n* + 1 heterogeneous vehicles is depicted in Figure 3, which is the main research object of this study. The primary objective of the CACC platoon control is to keep each CACC vehicle’s longitudinal motion following its predecessor with a Constant Time Gap (CTG). Among the controlled platoon, the inter-vehicle spacing is one of the main control variables, which is affected directly by the relative speed between the subject vehicle and the predecessor. In addition, the acceleration of the controlled vehicle is also one of the key factors that indirectly affects the inter-vehicle distance in the platoon. Typically, the velocity and acceleration can be detected by the onboard sensors, such as Radar, speedometer, accelerometer, and so on. In order to avoid a vehicle crash in the platoon, each vehicle in the CACC platoon is required to broadcast and report their Basic Safety Messages (BSMs), including position, velocity, and acceleration, periodically. According to the requirement of the National Highway Traffic Safety Administration and the Crash Avoidance Metrics Partnership, it is said that in future Vehicular Ad hoc Networks (VANETs), BSMs would be transmitted by all vehicles every 100 ms, to enhance cooperative driving and prevent rear-end crashes [26,27,28]. As Figure 4 displays, in the distributed communication structure, the predecessor–follower (PF) communication topology is one of the most widely-used V2V communication technologies, which is proved to be more resilient to communication failure compared with centralized communication topology [29].

Let us consider a Constant Time Gap (CTG) supported by wireless communication within a platoon. In a cooperatively controlled vehicle platoon, the car-following control of vehicle-*i* can be defined specifically by the system state of the predecessor vehicle. The objective of each vehicle is to keep pace with the preceding vehicle at a desired distance, the CACC controller (as described in [29]) considers a series of variables of vehicles, and the system state can be defined as a vector (*X_i_* = (*d_i_, v_i−1_, v_i_, a_i_*)), where *d_i_* is the gap or spacing between the preceding vehicle and the following vehicle. As Figure 4 displays, the spacing and spacing errors can be expressed as:

(5) $$d_{i}(t)=x_{i}(t)-x_{i-1}(t)-l_{i}$$

(6) $$\delta_{i}(t)=d_{i}(t)-h_{i}v_{i}(t)-d_{0}$$

where $l_{i}$ denotes the length of vehicle *i*; $x_{i}$ and $x_{i-1}$ represent the position of the *i-th* and *i−1-th* vehicle in the platoon, respectively. $\delta_{i}(t)$ denotes the spacing errors between the desired distance and the actual distance. $h_{i}$ is the headway time of the controlled vehicle, and $d_{0}$ represents the standstill minimal distance.

With the introduced vehicle platoon dynamics model, the feedback CACC control law [29] can be designed as:

(7) $$u_{i}(t)=f\{X_{i}(t-\Delta )\}=f\{(d_{i}(t-\Delta ),\;v_{i-1}(t-\Delta ),\;v_{i}(t),\;a_{i-1}(t-\Delta )\}$$

In Equation (7), f{} denotes the specific platoon control algorithm. It is notable that this control law is based on the V2V or V2I vehicular communication system. This state-feedback control law provides the relationship between the desired acceleration, *u_i_*, and the state vector, *X_i_*. Since the acceleration, velocity and position of the preceding vehicle are transmitted by the Vehicular Ad hoc NETwork, the inherent existing communication delay term Δ is contained in this control model. Furthermore, the control law with a communication delay can be specifically expressed as:

(8) $$u_{i}(t)=k_{a}\cdot a_{i-1}(t-\Delta )+k_{v}\cdot \left[v_{i-1}(t-\Delta )-v_{i}(t)\right]+k_{s}\cdot \delta_{i}(t-\Delta )$$

where $k_{a}$ represents a gain in the preceding vehicle’s acceleration; and $k_{v}$, $k_{s}$ is the gain of the velocity and the spacing difference between the following *i-th* vehicle and its preceding vehicle, respectively. In previous literature [29], the control model parameter was verified by numerical analysis. To maintain the CACC string stability, the parameter can take the values shown in Table 2.

### 2.3. Adaptive Cruise Platoon Control Strategy

Theoretically, compared with the Adaptive Cruise Controller (ACC), though the CACC controller can maintain string-stable vehicle-following behavior at smaller intervehicle distances, but CACC is vulnerable to communication impairments such as packet loss [29], in which case the CACC platoon has to degrade to conventional ACC. Meanwhile, the minimal intervehicle distance required for string-stable behavior is increased suddenly, and such degradation would potentially lead to traffic congestion, potential collisions, and even deteriorate the whole traffic flow stability. In this paper, one of the most difficult threats in CACC is considered, where V2V and V2I communication is assumed to completely fail. At that point, the autonomous vehicles can only sense information using their onboard sensors and then generate commands to the vehicle’s braking, steering, or acceleration systems. The widely used linear ACC controller for non-connected automated vehicles (NAVs) with the CTG policy [24] can be expressed as:

(9) $$u_{i}(t)=k_{v}\cdot \left[v_{i-1}(t-\xi )-v_{i}(t)\right]+k_{s}\cdot \delta_{i}(t-\xi )$$

In above ACC control model, ξ represents the sensor delay and actuator lag of the following vehicle; $k_{v}\cdot$ and $k_{s}$ are feedback gains on the speed error and gap error respectively. In this control model, the ACC parameters can be values like those in Table 3, which was verified in the literature [24] using theoretical derivation.

## 3. Safety Reinforced CACC Strategy

In this section, we will introduce the mechanism of the proposed safety-reinforced CACC system. The safety-reinforced CACC system incorporates both a dual branch control structure and the smooth transition algorithm to improve robustness and safety performance of the vehicle platoon. We first describe the proposed dual-branch control system, then we present how to switch smoothly between the two branches. Finally, we elaborate on the combined methods.

### 3.1. Safety Reinforced CACC Control Structure 

The main difference between the safety-enhanced platoon control strategy proposed in this paper and the regular CACC system is the dual branch control strategy (see Figure 4). The regular CACC is vulnerable to unreliable wireless communication due to high latency or packet loss [29], which may deteriorate platoon performance and cause collisions between vehicles [30]. To solve this problem, firstly, we proposed a dual-branch synthesis cooperative control structure, which is devoted to making up for the instability of the communication system. Then, a control algorithm is proposed to ensure that the system can smoothly transform between these two branches. The overall control procedures of the proposed SR-CACC scheme are summarized in the SR-CACC algorithm, which is specifically illustrated in the Algorithm 1.

To reduce the harmful effects of unreliable V2V communications and to enhance the platoon’s safety and stability, a feedforward control module was integrated into the Safety-Reinforced Cooperative Adaptive Cruise Control (SR-CACC) system. The feedforward control module is mainly developed to detect the status of the onboard vehicular communication system, which is one of the most noticeable features that differentiates it from conventional CACC systems and may have a significant influence on the cooperative platoon. When a failure in wireless communications is detected, the developed safety-enhanced SR-CACC system will have an alternative control strategy to maintain the platoon string-stable and avoid the platoon from falling into a disorderly out-of-control state. The sensor-based ACC controller is designed as the alternative strategy, which can operate without a wireless communication link.
**Algorithm 1** the SR-CACC algorithm**Input:** The length of simulation time *T*; the length of each step in the simulation *t_s_*; the number of vehicles in the platoon *N*; the leading vehicle driving profiles $\left(x_{0},v_{0}\;,a_{o}\right)$; **Output:**$\;\mathrm{driving}\;\mathrm{profiles}\;\mathrm{of}\;\mathrm{the}\;\mathrm{vehicle}\;\mathrm{in}\;\mathrm{the}\;\mathrm{SR}-\mathrm{CACC}\;\mathrm{platoon}\;(x_{i},v_{i},a_{i}$); 1: **Initialize:**
$x_{0}$$,\;k,\;v_{h}$(*·|k*); 2: **for** *k* = 1→ *T*/*t_s_* do 3:    Detect the communication status C_S_ = Ture or False, Whether the communication status has changed,  4:   Determine whether to excute the smooth transition module, and switch the control algorithm; 5:   **If** C_S_ = Ture 6:     $\mathrm{Solve}\;(8)\;\mathrm{to}\;\mathrm{compute}\;u_{i}^{*}$$(\text{·}|k)\;\mathrm{and}\;\mathrm{apply}\;u_{i}^{*}$(·|k) in vehicle i; Calculate         $v_{i}\;(\text{·}|k)\;\mathrm{and}\;x_{i}^{*}$(·|*k +* 1); 7:      $\mathrm{Transmit}\;\mathrm{the}\;\mathrm{BSM}\;\mathrm{message}\;(x_{i},v_{i},a_{i}$) it to the neighbor vehicle; 8:   **else** 9:     $\mathrm{Solve}\;(9)\;\mathrm{to}\;\mathrm{compute}\;u_{i}^{*}$$(\text{·}|k)\;\mathrm{and}\;\mathrm{apply}\;u_{i}^{*}$$\;(\text{·}|k)\;\mathrm{in}\;\mathrm{vehicle}\;i;\;\mathrm{Calculat}\;v_{i}$        $(\text{·}|k)\;\mathrm{and}\;x_{i}^{*}$(·|*k* + 1); 10:   **end if** 11: **end for** 12: $\mathrm{return}\;(x_{i},v_{i},a_{i}$)

The safety-reinforced cooperative adaptive cruise control system was mainly developed to reduce the risks of rear-end collision under the condition that the controlled vehicles failed to receive the information transmitted from their preceding vehicle and leading vehicle because of wireless communication malfunction, which may be caused by a physical barrier or unexpected extreme weather conditions. As shown in Figure 4 the flowchart illustrates the specific work process of the developed SR-CACC system. First, with the help of various vehicular equipment components, such as the speedometer, accelerometers, and accurate positioning differential GPS, the real-time dynamic driving data (position $x_{i}$, velocity $v_{i}$, and acceleration $a_{i}$) of the subject vehicles is collected and recorded on the onboard database system. This basic safety information (BSI) collected and recorded in the database has two main functions; one is to back up the driving data for users to query in the future and the other is to prepare to broadcast to platoon members, which is used to calculate desired accelerations ($u_{i}$) and maintain the string stability and avoid rear-end collision. 

Then, a real-time V2V communication status detection module added to the SR-CACC system will check the current communication status. Based on the obtained real-time communication status, the control module will decide the system enters which branch. Specifically speaking, if the following vehicle can obtain the preceding vehicle’s dynamic driving data (position $x_{i-1}$, velocity $v_{i-1}$, and acceleration $a_{i-1}$) through the V2V communication system, the SR-CACC system will execute the CACC control process, which will calculate the desired acceleration ($u_{i}$) and keep the vehicle platoon at a string-stable state. 

On the other hand, once the SR-CACC system has lost the wireless communication link, eventually, all the vehicles in the platoon will not receive the basic safety message (BSM) from the V2V communication system. To ensure the platoon’s stability and avoid rear-end crash accidents, the SR-CACC system has to transform the control process into the alternative ACC branch. In this branch, without the assistance of the wireless communication system, onboard sensors equipped on the autonomous vehicle, including Radar, LiDAR, and cameras, will measure the relative velocity and the distance with respect to the preceding vehicle. Based on these real-time driving data gathered by onboard sensors, the safety enhanced control system can adjust the acceleration of the host vehicle to maintain a desired headway time to the preceding vehicle. Meanwhile, to avoid constantly switching between CACC and ACC branches, the Smooth Transition Module is added to the improved system. The Smooth Transition Module will execute the conversion algorithm and suppress the frequent branch switching until the transition process is executed completely.

### 3.2. Smooth Transition Algorithm

From the above description of the flowchart, it is evident that these two branches correspond to the V2V communication-based CACC control algorithm and the sensor-based ACC algorithm, respectively, in this safety-reinforced CACC system. Though the sensor-based ACC control algorithm is applied as a supplementary alternative substitute strategy to improve the performance of the SR-CACC system, the inherent difference between these two strategies cannot be ignored. One of the most significant differences between these two algorithms is the headway time required to maintain string-stable behavior. According to recent research reports [15,27,28,29], the minimal string-stable time headway of the CACC system and the ACC system is about 0.5 s and 1.2 s, respectively, which means that the headway time required by these two control strategies is significantly different. Without proactive control measures to intervene in the conversion process, frequent switching behaviors may be triggered by stochastic packet loss and high latency, resulting in abnormal vehicle jitter behaviors. These abnormal shaking behaviors produced by the cooperatively controlled vehicles are very harmful and hazardous, which can even cause severe disturbances and deteriorate the string stability of the vehicle platoons. 

**Figure 5:**
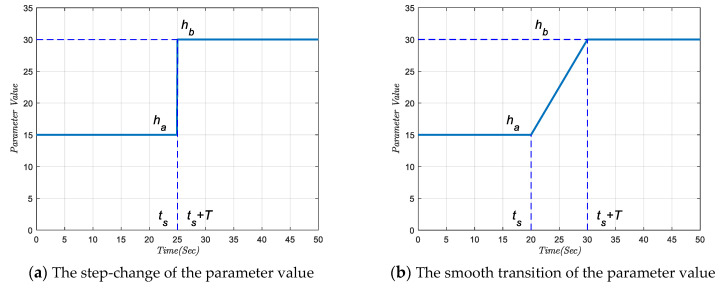
The working mechanism of smooth transition algorithm.

To ensure smooth switching between the two controlling strategies, we implemented a smooth transition algorithm in the SR-CACC system. Assuming that the buffer time of a transforming process is set as time T, we impose the linear change from the CACC parameter to the ACC one as:

(10) $$\hbar (t)=\hbar_{a}+\frac{\left(\hbar_{b}-\hbar_{a})(t-t_{s}\right)}{T}\;\mathrm{when}\;t\in [t_{s},t_{s}+T]$$

where $t_{s}$ represents the start of the transforming time point, at which point the SR-CACC system starts changing the inner control algorithm. $\hbar_{a}$ denotes the default parameter before the state transition; $\hbar_{b}$ is the desired parameter value after the state transition is completed. In addition, ℏ(t) denotes the temporary parameter value during the transforming process. The above linear smooth transition algorithm can be used to adjust all the jumping parameters, including the headway time $h_{i}$, velocity error gains $k_{v}$, gap error gains $k_{s}$. As shown in Figure 5, the proposed linear smooth transition algorithm is mainly devoted to eliminate the step jump of the inner system parameter. Thus, the proposed smooth transition algorithm can mitigate jerky vehicle behaviors in the SR-CACC system with an appropriate transition time.

## 4. Experiment and Simulation Results

This section will verify the effectiveness of the proposed SR-CACC system presented in Section 3 and investigate the platoon system performance through extensive simulation studies. The simulation experiment is developed based on the MATLAB/Simulink module. Furthermore, we will test the SR-CACC system without/with the smooth transition algorithm, respectively, to demonstrate the effectiveness of the proposed smooth transition algorithm. 

### 4.1. Design of the Simulation Experiment

We conducted the simulation experiment under a mixed traffic flow scenario, which is designed as shown in Figure 3. Initially, it was considered a heterogeneous platoon with one leading car and seven followers, which contains various vehicles, including cars, buses, and trucks. In the heterogeneous platoon, each vehicle has different inherent parameters, such as the mass, length, and frontal area of the vehicle. To simulate a more realistic traffic scenario, specific vehicle parameters are set as Table 4. Based on these parameters, the linearized vehicle longitudinal dynamic model is used in the following simulation experiments. In addition to this, as is shown in Figure 3, the vehicles in the platoon are connected with other members using the predecessor-follower communication topology, which is one of the most widely-used V2V communication technology.

**Figure 6:**
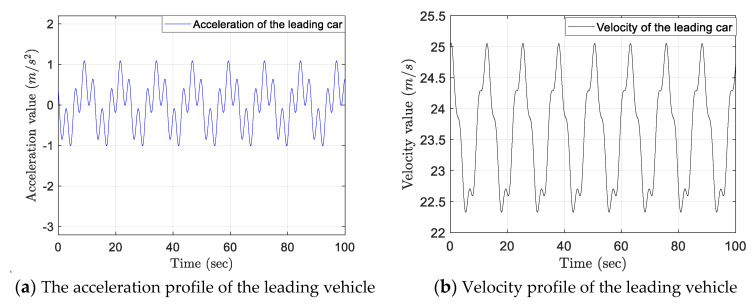
Acceleration and velocity profile of the leading vehicle.

To ensure the safety of the vehicles in the experiment, the maximum acceleration and minimal deceleration are limited within (${-3\;m/s}^{2}{,+2\;m/s}^{2}$) throughout the simulation experiment, which fulfills the requirements of the ISO standard 15622 for intelligent transport systems [30]. Moreover, the acceleration profile of the vehicle platoon leader is designed as shown in Figure 6a, which is a continuous stochastic fluctuation curve. Correspondingly, the leading vehicles’ longitudinal velocity profile is displayed in Figure 6b. To reproduce the real high-speed traffic scene, the leading platoon vehicle’s initial speed is set to 25 m/s. After that, as the acceleration fluctuates periodically, the longitudinal velocity of the leading vehicle will also fluctuate periodically, which ranges from about 22 m/s to 25 m/s throughout the experiment.

To verify the performance of the SR-CACC system under unreliable communication environments, firstly, an experimental cooperative platoon is set up with eight heterogeneous vehicles, which are all controlled using the proposed dual-branch SR-CACC system. Then, we consider the scenario where the vehicle platoon moves forward stably and suddenly suffers from an unexplained V2V communication failure. When the communication failure happens, all the vehicles in the platoon lose their wireless communication links instantaneously. At this point, the SR-CACC control system will switch its inner control algorithm to the ACC branch to continue maintaining the string stability of the platoon. In the designed experiment, to simulate the unexpected communication system failure, we actively turn off all vehicles’ onboard communication systems, and the experiment lasted for 40 s.

### 4.2. Simulation Experiment without the Smooth Transition

**Figure 7:**
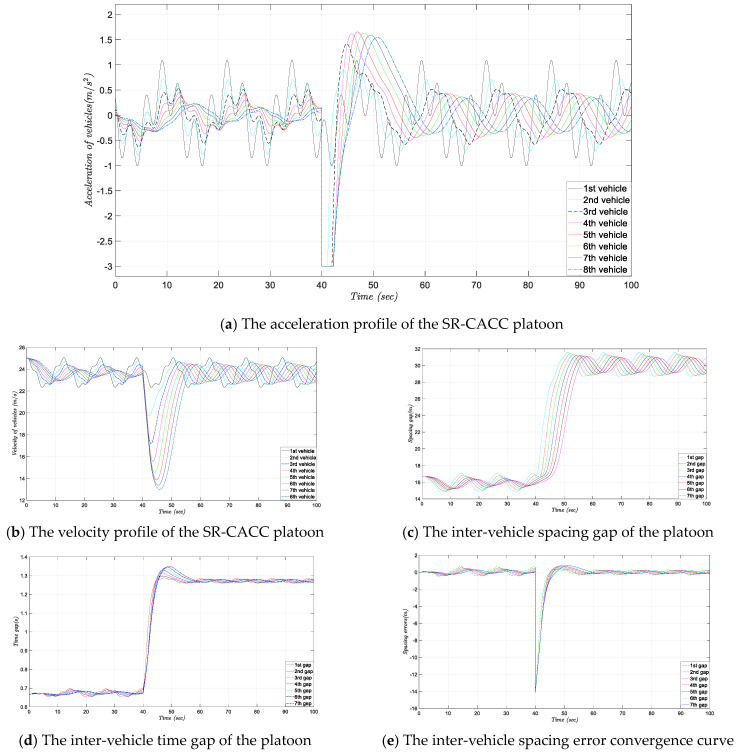
Simulation experiment results of the SR-CACC platoon without a smooth transition.

It is worth noting that, in this experiment, the smooth transition control module is not added in the primary SR-CACC system. Figure 7 displays the simulation experiment results under the designed scenario. More specifically, Figure 7a is the acceleration response curve of the platoon, which reflects the specific control output of each vehicle in the simulation experiment process. Subsequently, the velocity profile of the vehicles in the platoon is shown in Figure 7b, which reveals the real-time speed response of each vehicle during the experiment. After that, Figure 7c,d displays a diagram of the inter-vehicle distance gap and time gap, respectively, which reflect the relative distance between each vehicle in terms of time and space. Finally, the inter-vehicle spacing error convergence curve is shown in Figure 7e. The spacing error profile reveals the difference between the actual headway distance and the expected headway distance during the simulation experiment.

The experimental results indicate that when the simulation experiment progressed to about 40 s, the acceleration of the following vehicles has a significant step-change. From Figure 7a, we can observe that due to the communication failure is triggered at that time point, the accelerations of the following vehicles in the platoon step to the minimal deceleration boundary 3 m/s^2^ immediately. Moreover, because of the aggressive changes of the acceleration, as is shown in Figure 7b, the velocity of the following vehicle also fluctuates more drastically. Meanwhile, Figure 7e also reveals that the inter-vehicle distance error of the SR-CACC system exhibited a rapid increase when the communication failure occurs, which may be one of the main causes of the controlled vehicles’ acceleration sharp jitter. Finally, from Figure 7c can know, to guarantee the string stability of the vehicle platoon, the inter-vehicle spacing distance converged from around 15 m to about 30 m. Correspondingly, the inter-vehicle headway time of the platoon transformed from 0.67 s to about 1.25 s, which can be observed in Figure 7d. 

### 4.3. Simulation Experiment with the Smooth Transition 

**Figure 8:**
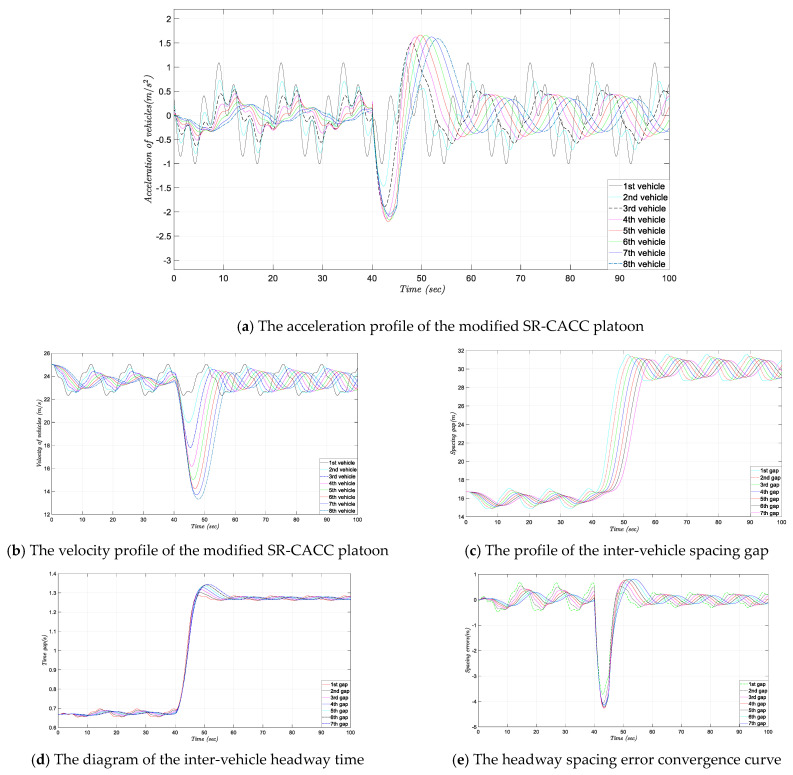
Simulation experiment results of the SR-CACC platoon with a smooth transition.

To test the performance of the improved control strategy, which integrated the smooth transition algorithm in the dual-branch SR-CACC system, we conducted the simulation experiment again, using the same leader acceleration and speed profile. Different from the original SR-CACC system, when the control system is suffering from communication failure, the improved SR-CACC system will execute the smooth transition process before switching into the ACC strategy. In this simulation experiment, the transition process time is set as 5 s, which means that from 0 to 40 s, the platoon is controlled by the inner CACC strategy, and from 40 to 45 s is the smooth transition process. After that, the system enters the ACC control branch. The simulation experiment results are shown in Figure 8. Similar to the previously obtained experiment results, the acceleration profile, velocity diagram, spacing gap curve, headway time graph, and spacing error convergence curve are displayed in Figure 8a–e, respectively.

### 4.4. Discussion of the Experiment Results 

As shown in Figure 8, compared to the SR-CACC system without the smooth transition module, vehicles controlled by the improved SR-CACC system respond to wireless communication failure more effectively. Further, comparing Figure 7a and Figure 8a, the acceleration response curve under the smooth transition strategy is much smaller in amplitude. Based on these experiments, we can see that the absolute value of acceleration reduced from 3 m/s^2^ to about 2.3 m/s^2^, which means that, under our designed experimental scenario, the amplitude is reduced by about 23.3%. This significant reduction indicates that the aggressive braking and violent accelerating behaviors have been suppressed effectively in the smooth transition strategy. Moreover, the comparison experiments also illustrate that the continuous transition process has replaced the former accelerations’ discontinuous oscillation. Therefore, it is recognized that the accelerations’ discontinuous oscillation is one of the dangerous behaviors that may undermine the safety and stability of the vehicle platoon. Meanwhile, as shown in Figure 8b–d, the effective acceleration profile generates a more stable velocity, inter-vehicle spacing gap, and headway time profile. Finally, by comparing the headway spacing errors shown in Figure 7e and Figure 8e, it is evident that the distance error has been significantly suppressed in the improved SR-CACC system.

## 5. Conclusion and Future Work

In this paper, we proposed a safety-reinforced cooperative adaptive cruise control strategy to avoid rear-end collision accidents and guarantee the string stability of the heterogonous platoon. Unlike the regular CACC system, the safety enhanced platoon control system is embedded with a dual-branch control strategy, which is devoted to mitigating the safety hazards caused by unexpected communication failure. When the fatal wireless communication failure is detected and confirmed in our designed system, the SR-CACC system will automatically activate the alternative sensor-based ACC control strategy to avoid the vehicle platoon from falling into uncontrolled chaos. However, in the simulation experiment, we observed that the inherent difference between these two strategies can cause abnormal oscillation, which may undermine the safety performance of the vehicle platoon. To solve this problem and make the switching process smooth, a linear smooth transition algorithm is embedded in the SR-CACC system. Then, to verify the effectiveness of the proposed SR-CACC system, we conducted a series of simulation experiments under the designed experiment scenario. The obtained experimental results demonstrate that the proposed SR-CACC system with a smooth transition module can effectively improve the system’s ability to resist communication failures. Furthermore, further experiments reveal that the proposed smooth transition algorithm can effectively suppress aggressive braking and violent accelerating behaviors. 

For future work, the proposed SR-CACC system can be extended in a few important directions. In this paper the liner smooth transition algorithm is proposed and applied in the feed forward module to mitigate abnormal acceleration jerk. More complicated no-liner smooth transition algorithm can be investigated in future research. Further, more research are need to explore more sophisticated communication environments. To simulate the complicated V2V communication, a network simulator like OMNeT ++ could be introduced in future research. Finally, more experimental scenarios need to be designed and investigated to deepen further research, which is essential to ensure societal acceptance and trust this novel SR-CACC system.

## Figures and Tables

**Table 1:** Parameters notations of the vehicle dynamic model.

Symbol	Definition	Unit	Example Value
m	Mass of the vehicle	(kg)	1555
g	Gravitational constant	(${m/s}^{2}$)	9.81
ρ	Air density	(${\mathrm{kg}/m}^{2}$)	1.20
$C_{d}$	Aerodynamic drag coefficient	-	0.335
*A_F_*	The frontal area of the vehicle	($m^{2}$)	2.3

**Table 2:** Parameters value of the CACC controller.

Symbol	Definition	Value
$k_{a}\cdot$	Gains of the acceleration	0.6
$k_{v}$	Gains of the speed errors	0.4
$k_{s}$	Gains of the distance errors	0.2
h	The headway time gap (s)	0.6
Δ	Communication time delay (ms)	100

**Table 3:** Parameters value of the ACC controller.

Symbol	Definition	Value
$k_{v}$	Gains of the speed errors	0.8
$k_{s}$	Gains of the distance errors	0.6
h	The headway time gap (s)	1.2
ξ	Sensor delay and actuator lag (ms)	200

**Table 4:** Vehicles’ parameters in the heterogeneous platoon.

Vehicles Type	Mass (kg)	Length (m)	Frontal Area (m^2^)
Car	[1500, 3000]	[3, 4.5]	[1.8, 2.4]
Truck	[4000, 8000]	[4.5, 8]	[2.6, 4.5]
Bus	[3500, 5000]	[4, 6]	[2.4, 4.2]

## Data Availability

No new data were created or analyzed in this study. Data sharing isnot applicable to this article.
